# Predictors of telemedicine use during the COVID-19 pandemic in the United States–an analysis of a national electronic medical record database

**DOI:** 10.1371/journal.pone.0269535

**Published:** 2022-06-29

**Authors:** Sameed Ahmed M. Khatana, Lin Yang, Lauren A. Eberly, Howard M. Julien, Srinath Adusumalli, Peter W. Groeneveld

**Affiliations:** 1 Division of Cardiovascular Medicine, Perelman School of Medicine, University of Pennsylvania, Philadelphia, Pennsylvania, United States of America; 2 Penn Cardiovascular Outcomes, Quality, & Evaluative Research Center, Perelman School of Medicine, University of Pennsylvania, Philadelphia, Pennsylvania, United States of America; 3 The Leonard Davis Institute of Health Economics, University of Pennsylvania, Philadelphia, Pennsylvania, United States of America; 4 Penn Cardiovascular Center for Health Equity and Social Justice, Perelman School of Medicine, University of Pennsylvania, Philadelphia, Pennsylvania, United States of America; 5 Penn Medicine Center for Health Care, University of Pennsylvania, Philadelphia, Pennsylvania, United States of America; 6 Division of General Internal Medicine, Perelman School of Medicine, University of Pennsylvania, Philadelphia, Pennsylvania, United States of America; 7 Center for Health Equity Research and Promotion, Michael J. Crescenz Veterans Affairs Medical Center, Philadelphia, Pennsylvania, United States of America; University of Utah, UNITED STATES

## Abstract

Telemedicine utilization increased significantly in the United States during the COVID-19 pandemic. However, there is concern that disadvantaged groups face barriers to access based on single-center studies. Whether there has been equitable access to telemedicine services across the US and during later parts of the pandemic is unclear. This study retrospectively analyzes outpatient medical encounters for patients 18 years of age and older using Healthjump–a national electronic medical record database–from March 1 to December 31, 2020. A mixed effects multivariable logistic regression model was used to assess the association between telemedicine utilization and patient and area-level factors and the odds of having at least one telemedicine encounter during the study period. Among 1,999,534 unique patients 21.6% had a telemedicine encounter during the study period. In the multivariable model, age [OR = 0.995 (95% CI 0.993, 0.997); p<0.001], non-Hispanic Black race [OR = 0.88 (95% CI 0.84, 0.93); p<0.001], and English as primary language [OR = 0.78 (95% CI 0.74, 0.83); p<0.001] were associated with a lower odds of telemedicine utilization. Female gender [OR = 1.24 (95% CI 1.22, 1.27); p<0.001], Hispanic ethnicity or non-Hispanic other race [OR = 1.40 (95% CI 1.33, 1.46);p<0.001 and 1.29 (95% CI 1.20, 1.38); p<0.001, respectively] were associated with a higher odds of telemedicine utilization. During the COVID-19 pandemic, therefore, utilization of telemedicine differed significantly among patient groups, with older and non-Hispanic Black patients less likely to have telemedicine encounters. These findings are relevant for ongoing efforts regarding the nature of telemedicine as the COVID-19 pandemic ends.

## Introduction

The coronavirus disease 2019 (COVID-19) pandemic has transformed much of the US healthcare landscape. One of the most dramatic changes has been the widespread usage of telemedicine services for patient-provider interactions. Prompted by regulatory changes by the Centers for Medicare and Medicaid Services (CMS), many outpatient providers in the US adopted telemedicine services for the first time. Understanding how patients utilize telemedicine services and whether there has been equitable availability is important for providers and payers to maximize the benefit of telemedicine, particularly as the US emerges from the pandemic.

With widespread stay-at-home orders implemented across many areas in the US at the onset of the COVID-19 pandemic, and with subsequent curtailing of elective procedures and services, CMS authorized payment for outpatient, hospital, and other medical visits provided through audio and visual technologies at the same rate as in-person visits [1]. Many commercial insurance providers also authorized payments for telemedicine services [2]. In one study of commercially insured patients, by June 2020, telemedicine encounters comprised close to 20% of all outpatient encounters, however significant variation existed between states [3]. Because novel medical services and technologies are often not equally available, and as a consequence of the well-documented digital divide, there has been concern raised that certain historically disadvantaged groups may not have similar access to telemedicine services as more advantaged groups. In two studies from a single health system that analyzed encounters from early in the COVID-19 pandemic, older patients, women, patients residing in lower-income areas, and racial and ethnic minority patients had lower utilization of telemedicine services [4,5]. Whether disparities in access to telemedicine services exist across different healthcare providers, systems, and regions, particularly later in the course of the pandemic, is uncertain.

To address these questions, using a national database of electronic medical records (EMR), we examined trends in outpatient telemedicine utilization from March through December 2020 and assessed whether utilization varied between different patient groups.

## Methods

All data utilized were de-identified and collected as part of routine healthcare provision. The COVID19 Research Database project was considered exempt by the Western Institutional Review Board (Puyallup, WA) and informed consent was waived.

### Patient data

Data on medical encounters was obtained from Healthjump, a data management platform that allows for interoperability across different EMR vendors such as Cerner, Epic, Allscripts and others. For healthcare organizations utilizing Healthjump, this allows for standardization of records across platforms. A full list of EMR vendors for which Healthjump can extract data from is available on the Healthjump website [6]. The Healthjump database contains EMR data for approximately 40 million unique patients, in all 50 US states and Washington D.C., updated daily. Extracted data for this analysis includes data on encounters, comorbidities, medications, and demographics. Patient residence at the 3-digit ZIP code level was available. Healthjump has previously been used to investigate patterns of healthcare utilization during the COVID-19 pandemic [7]. The database was made available through the COVID19 Research Database project, which is a public-private initiative providing pro-bono access to multiple commercial databases for researchers to investigate questions related to the COVID-19 pandemic [8].

### Social vulnerability index

To assess area-level factors that make a community more vulnerable to the adverse effects of a health disaster, we used the Centers for Disease Control and Prevention’s (CDC) Social Vulnerability Index (SVI), which has been associated with disparities in health care access and outcomes in several studies [9–12]. The SVI comprises 15 US Census-derived variables grouped into 4 categories: 1) socioeconomic, 2) household composition and disability, 3) minority status and language, and 4) housing and transportation. Each community is ranked on a scale of 0 to 100 for each factor, and the SVI is the average of these ranks. A higher value for the SVI, or each of its component indices, indicates a greater degree of social vulnerability to a health disaster. We obtained ZIP code tabulation area (ZCTA) level data for each of the 15 factors that compose the SVI using the 2019 version of the US Census Bureau’s 5-year American Community Survey. After, first using a cross-walk of ZCTA to 5-digit ZIP code, data were then aggregated to the 3-digit ZIP code level and the SVI (and each of the 4 component indices) was calculated. Details regarding the individual factors that comprise the SVI are included in Table 1 in S1 Appendix.

The proportion of residents living in rural areas was obtained from the 2010 US Census. An area was designated as rural if it was not classified as an urban area. Urban areas comprise any incorporated place or territory with at least 2,500 residents, based on US census criteria [13].

### Population

All individuals 18 years of age and older who had an outpatient encounter in the Healthjump database, between March 1, 2020 and December 31, 2020 were included. Outpatient encounters were identified using Medicare’s list of outpatient Common Procedural Terminology (CPT) codes which have been used in prior studies to identify such encounters (Table 2 in S1 Appendix) [3,14]. These codes exclude those that are for settings other than outpatient ambulatory care such as the emergency department.

### Primary outcome

The primary outcome was whether a patient had at least one telemedicine encounter during the study period. After identifying all outpatient encounters, telemedicine encounters were identified using the CPT codes 99441–99443 (audio only) or if they had the modifier codes GT, GQ, or 95 (audio-video). We did not analyze audio and audio-video visits separately in the primary analysis due to previously noted concerns about misclassification of specific telemedicine type using CPT codes [14]. Additionally, as patient’s health insurance type is not available, we are unable to identify patients who may be insured through payers such as Medicare that specifically require documentation of codes differentiating between audio-only and video visits. However, video visits were analyzed in an exploratory analysis as stated below.

### Statistical methods

We first identified all unique patients in the Healthjump database who had an outpatient encounter during the study period. Patients who had least one telemedicine encounter during the study period were in the telemedicine group and patients who had no telemedicine encounters in the non-telemedicine group. We then compared demographic and clinical comorbidities between these two groups using Student’s t-test for continuous variables and chi-square tests for discrete variables. We also calculated the proportion of patients with a telemedicine encounter at the 3-digit ZIP code of residence level and then compared these based on quartiles of the SVI at the 3-digit ZIP code level.

To determine the association of patient and area-level characteristics with the odds of having a telemedicine encounter, using a generalized linear mixed effects framework, we fit a logistic regression model with at least one telemedicine encounter during the study period as the primary outcome, and the following covariates: age, gender, race/ethnicity [Hispanic (any race), non-Hispanic Black, non-Hispanic Other, and non-Hispanic White], primary language (English vs. non-English), indicator variables for 39 medical comorbidities (based on the Elixhauser comorbidity index), the proportion of rural residents living in the 3-digit ZIP code of residence, and the socioeconomic, household composition and disability, minority status and language, and housing and transportation components of the SVI for the 3-digit ZIP code of residence [15]. The model also included random intercepts for medical provider (of first telemedicine encounter for the telemedicine group and first non-telemedicine encounter for the non-telemedicine group) to account for clustering of patients who received care from the same medical provider. Variable selection was based on previous studies suggesting differences in telemedicine utilization based on demographic variables such as age, gender, race/ethnicity and language [4,5]. Patient level medical comorbidities were included as patients with a higher degree of comorbidities may have a differential utilization of telemedicine services. Area level measures of socioeconomic status and rurality were included, as previous studies suggest that these factors may influence telemedicine access and utilization [16,17]. In a secondary, exploratory analysis, limited to patients who had at least one telemedicine encounter during the study period, the primary logistic regression model was refit with at least one video telemedicine (vs. no video telemedicine) as the outcome of interest.

To account for missing data for race/ethnicity and language, we used multiple imputation using fully conditional specification, with 10 imputations, using the following variables: age, gender, 39 comorbidity indicator variables, and the following 3-digit ZIP code level variables: proportion of residents who were Hispanic (any race), non-Hispanic Asian, non-Hispanic Black, non-Hispanic White, non-English speaking, with income below the poverty level, and living in a rural area.

Multicollinearity between variables included in the primary regression model was assessed by calculating a variance inflation factor value for each variable. A variance inflation factor value >5 was considered to be indicative of significant multicollinearity [18,19].

Results of the analysis are presented as means and standard deviations (SD) or 95% confidence intervals or medians and interquartile ranges (IQR) as indicated. P-values <0.05 were considered statistically significant. All analyses were conducted using SAS version 9.4.

## Results

A total 1,999,534 unique patients had an outpatient encounter in the Healthjump database between March 1 and December 31, 2020. The study population had a mean age of 52.4 (SD = 18.2) years with 29.6% of the population older than 64 years of age. Women constituted 58.2% of the population. Race and ethnicity data were missing for 36.7% of the study population and language data were missing for 32.3% of the population. Among patients with data available, 20.8% were Hispanic (any race), 13.8% were non-Hispanic Black, 14.8% were non-Hispanic other race, and 50.6% were non-Hispanic White. Among patients with data available, English was the primary language for 82.5%. Among patients whose primary language was not English, Spanish was the primary language for 21.2% and other languages were the primary language for the remaining. The proportion of individuals in each sub-group of race/ethnicity and language after multiple imputation are listed in Table 3 in S1 Appendix. Among the study population, 7.8% of patients resided in the Midwest, 10.9% in the Northeast, 54.2% in the South, and 27.0% in the West US Census Region. Patients had encounters with a total of 5,696 unique healthcare providers. The median number of unique patients among these providers was 159 (IQR 25–524).

Of the included patients, 21.6% (432,634 patients) had at least one telemedicine encounter, and 78.4% (1,566,900) had no telemedicine encounters over the study period (Table 1). There were a total of 891,507 telemedicine and 4,335,444 non-telemedicine encounters during the study period. Patients in the telemedicine group had a higher mean number of total outpatient encounters compared to the non-telemedicine group [4.1 (SD = 4.2) vs. 2.2 (SD = 2.2) respectively, p<0.001]. Among the telemedicine group, the mean number of telemedicine encounters was 2.1 (SD = 2.6). Patients in the telemedicine group had a higher mean age compared to the non-telemedicine group [53.0 (SD = 17.7) vs. 52.2 (SD = 18.3) years respectively, p<0.001] and were more likely to be women compared to the non-telemedicine group [62.6% vs. 57.0% respectively, p<0.001]. Among patients with available data, a higher proportion of the telemedicine group were Hispanic (any race), non-Hispanic Black, or non-Hispanic other race compared to the telemedicine group [Hispanic (any race): 29.1% vs. 18.5%, non-Hispanic Black: 13.9% vs. 13.7%, non-Hispanic other race 16.6% vs. 14.3% respectively, p<0.001]. A lower proportion of the telemedicine group had English as their primary language compared to the non-telemedicine group (75.7% vs. 84.4% respectively, p<0.001). The mean number of comorbidities (maximum of 39) was significantly higher in the telemedicine group compared to the non-telemedicine group [1.5 (SD = 1.5) vs. 0.9 (SD = 1.2), p<0.001]. Distribution of medical comorbidities in each group are included in Table 4 in S1 Appendix. There were also significant differences in the region of residence of included patients among the two groups based on US Census Regions. A greater proportion of patients in the telemedicine group resided in the Northeast and West than in the non-telemedicine group (Northeast: 15.8% vs. 9.6%, West: 33.3% vs. 25.3% respectively, p<0.001).

**Table 1:** Patient characteristics by telemedicine utilization.

	Any telemedicine encounter ^a^	No telemedicine encounter ^a^
Number of unique patients	432,634	1,566,900
Number of encounters (Mean, SD)	4.1 (SD = 4.2)	2.2 (SD = 2.2)
Age [years, Mean (SD)]	53.0 (17.7)	52.2 (18.3)
Female	270,849 (62.6)	893,166 (57.0)
Male	161,785(37.4)	673,734 (43.0)
**Race/ethnicity**		
Hispanic (any race) ^b^	80,893 (29.1)	182,548 (18.5)
Non-Hispanic Black ^b^	38,618 (13.9)	135,607 (13.7)
Non-Hispanic other race ^b^	46,022 (16.6)	141,504 (14.3)
Non-Hispanic White ^b^	112,003 (40.4)	528,787 (53.5)
Missing race/ethnicity data	155,098 (35.8)	578,454 (36.9)
**Primary language**		
Primarily English speaking ^b^	218,766 (75.7)	898,071 (84.4)
Primarily non-English speaking ^b^	70,230 (24.3)	166,548 (15.6)
Missing language data	143,638 (33.2)	502,281 (32.1)
Number of comorbidities (Mean, SD) ^c^	1.5 (1.5)	0.9 (1.2)
**Patient residence by US Census Regions**		
Northeast	12,527 (2.9)	143,491 (9.2)
Midwest	68,254 (15.8)	150,182 (9.6)
South	207,829 (48.0)	876,915 (56.0)
West	144,024 (33.3)	396,312 (25.3)

Results are presented as number of patients and percentage of patients in group (telemedicine or non-telemedicine), unless otherwise indicated. All differences between the two groups are statistically significant at p<0.001.

a. Any telemedicine encounter includes patients with at least one telemedicine outpatient medical encounter from March 1, 2020 to December 31, 2021 and no telemedicine encounter indicates no telemedicine outpatient encounters during this period.

b. Proportion of patients with data available. Proportions of patients in each race/ethnicity and language sub-group after multiple imputation are listed in Table 3 in S1 Appendix.

c. Proportion of patients in each group with individual medical comorbidities listed in Table 4 in S1 Appendix.

**Fig 1:**
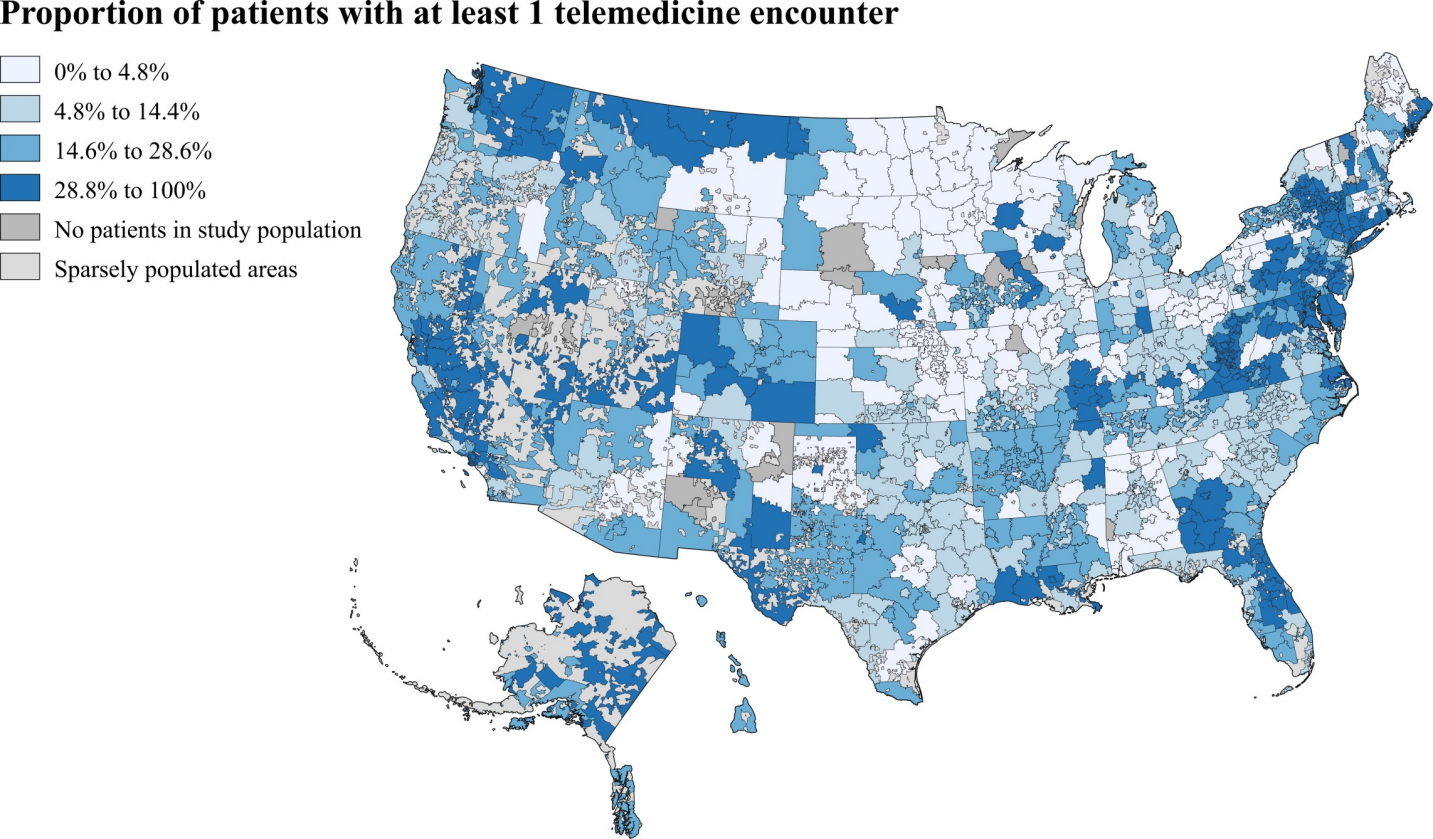
Proportion of patients with a telemedicine encounter (from March to December 2020) by 3-digit ZIP code. Figure based on 2019 ZIP code tabulation area shapefile created by US Bureau and Helathjump data [20]. Map projection based on modification of the US National Atlas Equal Area coordinate reference system. Proportion of patients in the study population with at least one telemedicine encounter during the study period in each 3-digit ZIP code divided into quartiles. Areas with no ZIP code tabulation area code provided by the US Census Bureau include sparsely populated areas and areas such as military facilities.

**Fig 2:**
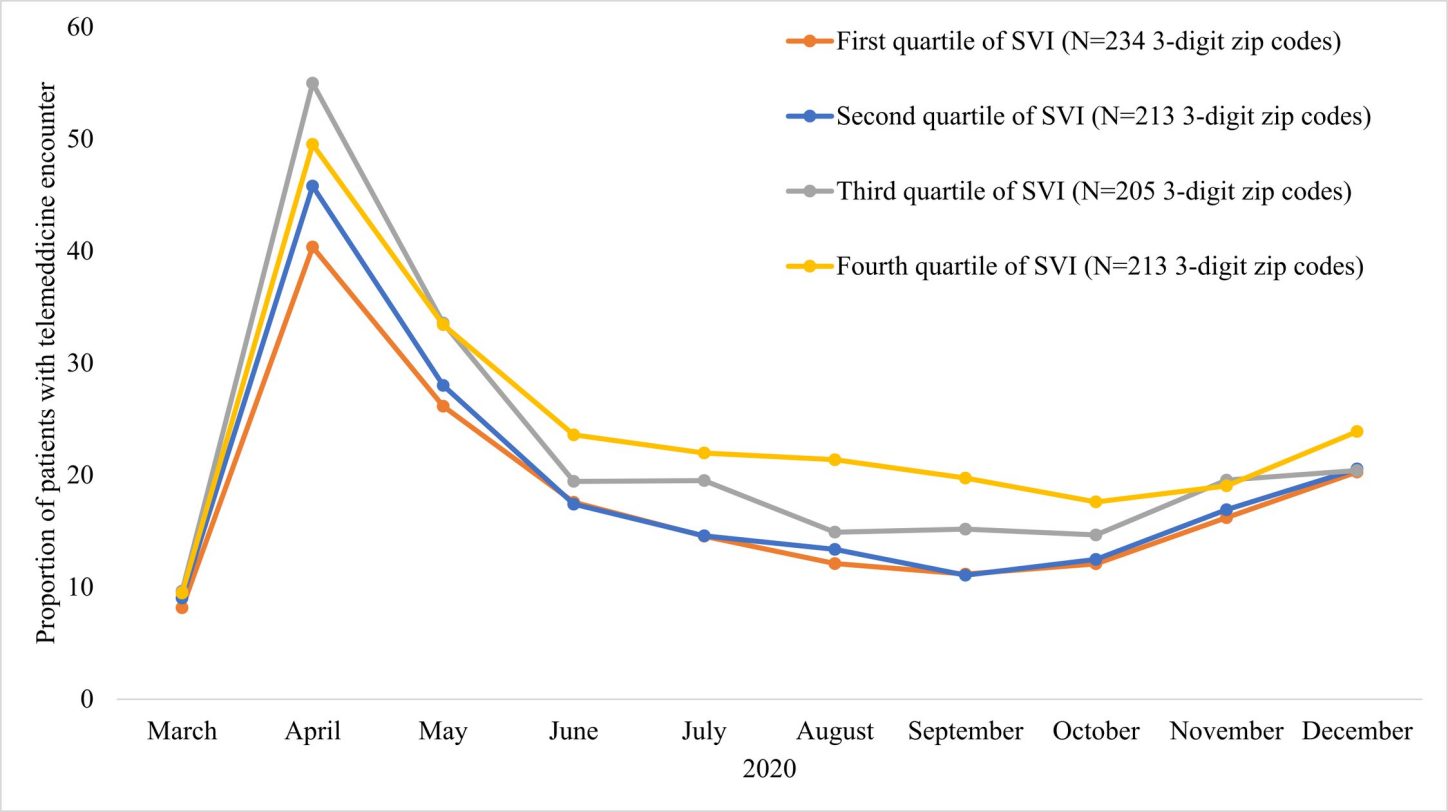
Monthly proportion of unique patients with outpatient telemedicine encounter from March to December 2020 by Social Vulnerability Index of 3-digit ZIP code of residence. Social Vulnerability Index (SVI) based on Centers for Disease Control and Prevention methodology [9]. A higher value of SVI indicates greater degree of social vulnerability of an area to a health disaster. Patients with telemedicine encounters in one month, not included in calculation for subsequent months.

Patients resided in a total of 865 3-digit ZIP codes across the US (Fig 1). Among patients living in 3-digit ZIP codes in the lowest quartile for SVI (least socially vulnerable) the proportion with a telemedicine encounter was 17.8%. The proportion of patients with a telemedicine encounter was 19.3% in the second quartile and 23.1% in the third quartile. For patients living in the highest quartile (most socially vulnerable) 3-digit ZIP codes, the proportion with a telemedicine encounter was 24.9% (p<0.001 for all pair-wise comparisons between the first quartile and each of the other quartiles). Monthly proportions of patients with a telemedicine encounter followed a similar temporal trend in all quartiles, with the highest proportion among patients living in the most socially vulnerable 3-digit ZIP codes throughout most of the study period (Fig 2). The monthly proportion of unique patients with telemedicine utilization and the proportion of total outpatient encounters conducted through telemedicine for the total study population are displayed in Figs 1 and 2 in S1 Appendix.

In the multivariable mixed effects logistic regression model, after accounting for differences in medical comorbidities and characteristics of the 3-digit ZIP code of patient residence, the following patient-level factors were associated with a lower odds of having at least one telemedicine encounter in the study: increasing age [OR = 0.995 (95% CI 0.993 to 0.997); p<0.001], being non-Hispanic Black compared to non-Hispanic White [OR = 0.88 (95% CI 0.84 to 0.93); p<0.001], and having English as primary language compared to non-English [OR = 0.78 (95% CI 0.74 to 0.83); p<0.001] (Table 2). The following patient-level factors were associated with a higher odds of having a telemedicine encounter: female compared to male gender [OR = 1.24 (95% CI 1.22 to 1.27); p<0.001], being Hispanic (any race) or non-Hispanic other race compared to non-Hispanic White [OR = 1.40 (95% CI 1.33 to 1.46);p<0.001 and OR = 1.29 (95% CI 1.20 to 1.38); p<0.001, respectively]. Among area-level covariates included in the multivariable model, a higher value of the housing and transportation subcomponent of the SVI was associated with a lower odds of having a telemedicine encounter [OR = 0.999 (95% CI 0.997 to 0.999); p = 0.04]. Living in an area with a higher socioeconomic subcomponent of the SVI was associated with a higher odds of having a telemedicine encounter [OR = 1.01 (95% CI 1.00 to 1.02); p = 0.02]. Odds ratios for indicators for each of the included comorbidities are included in Table 5 in S1 Appendix. Results from a complete case version of the primary model, which excludes patients with missing race/ethnicity and language data, are presented in Table 6 in S1 Appendix. Tests for multicollinearity in the model did not reveal a significant level of multicollinearity in any included covariate (Table 7 in S1 Appendix).

**Table 2:** Multivariable mixed effects logistic regression model with any outpatient telemedicine encounter as outcome.

	Odds Ratio (95% CI)	p-value
Age	0.995 (0.993, 0.997)	<0.001
Female	1.24 (1.22, 1.27)	<0.001
Non-Hispanic White	**Reference**	
Hispanic (Any race)	1.40 (1.33, 1.46)	<0.001
Non-Hispanic Black	0.88 (0.84, 0.93)	<0.001
Non-Hispanic other race	1.29 (1.20, 1.38)	<0.001
Primarily non-English speaking	**Reference**	
Primarily English speaking	0.78 (0.74, 0.83)	<0.001
Proportion of residents living in rural areas	0.99 (0.98, 1.00)	0.10
Socioeconomic index ^a^	1.01 (1.00, 1.02)	0.02
Household composition and disability index ^a^	1.00 (0.99, 1.00)	0.44
Minority status and language index ^a^	0.99 (0.98, 1.01)	0.24
Housing type and transportation index ^a^	0.999 (0.997, 0.999)	0.04

a. Sub-component of the CDC Social Vulnerability Index (SVI) based on Centers for Disease Control and Prevention methodology [9]. Higher value of SVI sub-component indicates greater degree of social vulnerability of an area to a health disaster.

Coefficients for indicator variables for medical comorbidities included in Table 5 in S1 Appendix.

In the exploratory analysis of video telemedicine visits, among the 891,507 patients with any telemedicine encounter over the study period, 125,644 individuals (14.1%) had at least one video encounter. In the multivariable mixed effects logistic regression model with any video telemedicine encounter vs. no video telemedicine encounter as the primary outcome (among individuals with any telemedicine encounter) the following patient-level factors were associated with a lower odds of having at least one video telemedicine encounter: increasing age [OR = 0.975 (95% CI 0.97 to 0.98); p<0.001], being non-Hispanic Black compared to non-Hispanic White [OR = 0.68 (95% CI 0.63 to 0.74); p<0.001], and being Hispanic (any race) compared to non-Hispanic White [OR = 0.56 (95% CI 0.52 to 0.61); p<0.001] (Table 8 in S1 Appendix). The following patient-level factors were associated with a higher odds of having a video telemedicine encounter: female compared to male gender [OR = 1.03 (95% CI 1.001 to 1.07); p = 0.043], being non-Hispanic other race compared to non-Hispanic White [OR = 1.37 (95% CI 1.16 to 1.60);p<0.001], and having English as primary language compared to non-English [OR = 2.05 (95% CI 1.82 to 2.31); p<0.001].

## Discussion

During the first 10 months of the COVID-19 pandemic in the US from March through December 2020, in a national database including outpatient medical encounters of approximately 2 million unique patients, 21.6% of patients received at least one telemedicine encounter. There were significant differences in the receipt of telemedicine encounters, with older age and non-Hispanic Black race associated with a significantly lower odds of having a telemedicine encounter. Female gender, and Hispanic ethnicity were associated with a higher odds of having a telemedicine encounter.

Prior to the COVID-19 pandemic, as demonstrated in a recent study of commercially insured patients, reimbursed telemedicine services in the US based on administrative claims data were non-existent [3]. To minimize infection risk due to the pandemic, as well as accommodate diversion of resources to COVID-19 related healthcare, CMS initially expanded payment for telemedicine services on a temporary basis in March [21]. Beyond Medicare, commercial payors and states have also expanded coverage for telemedicine encounters [2]. What role telemedicine plays in outpatient care as the pandemic ends is still unclear, with different availability and payment strategies being discussed by groups such as Medicare Payment Advisory Commission [22]. However, if telemedicine continues to play a large role in the post-pandemic era, understanding the disparities in access by different groups is important in ensuring its equitable availability.

In this analysis, compared to non-Hispanic White patients, non-Hispanic Black patients had a lower odds of having a telemedicine encounter. Although, a recent internet-based survey during the COVID-19 pandemic found that Black participants were more likely to have used telemedicine because of the pandemic, this study did not account for differences in the burden of medical comorbidities between participants [23]. Another study of patients in New York City during the pandemic found that after accounting for patient comorbidities and other factors, Black patients had a lower odds of receiving telemedicine care [24]. Although the current analysis did not investigate differences in types of telemedicine modalities used, disparities between non-Hispanic Black and non-Hispanic White patients may represent previously documented disparities in access to broadband internet for video telemedicine visits [25,26]. However, it is possible that there may also be a lower patient preference for telemedicine visits compared to inpatient visits among non-Hispanic Black patients. A previous study of the acceptability of telemedicine among Black and Hispanic individuals noted a greater degree of concern about confidentiality, privacy, and not having a medical provider physically present among Black individuals [27]. These concerns may be rooted in part in the long history of racism and discrimination experienced by Black individuals in the US in their interactions with the health care system. Therefore, to provide equitable access to telemedicine services, providers and administrators will need to understand the unique concerns of individuals and communities and ensure adequate quality of telemedicine encounters.

The lower rate of utilization of telemedicine by men, compared to women, mirrors several studies that have demonstrated a lower rate of health seeking behaviors among men [28–31]. Some prior studies have also suggested that men may be less likely to be offered medical advice and also receive less of a medical provider’s time during an encounter compared to women [32,33]. Our analysis also demonstrated a significant association between older age and a lower odds of receiving a telemedicine encounter. Several factors, including difficulties with technologies, lower rates of cell-phone and smartphone usage, as well as hearing or visual impairment may play a role in this [34]. A greater aversion to telemedicine compared to in-person visits among older individuals has been noted previously due to concerns about the quality of telemedicine encounters [35]. Given the obvious benefit of telemedicine encounters in the elderly, particularly those who may be frail or have transportation issues, expanding access in this population, while ensuring adequate quality of care provided, is likely to have significant benefits.

Our analysis also noted that Hispanic patients (of any race) compared to non-Hispanic White patients and primarily non-English speakers compared to English speakers had higher odds of telemedicine utilization. Some previous single center studies have noted lower utilization of telemedicine among Hispanic patients [36,37]. However, another study noted that Hispanic patients had a higher rate of having video telemedicine encounters than non-Hispanic White patients [38]. Additionally, one study of predominantly Hispanic individuals at a community health event found that approximately 80% of participants exhibited positive attitudes towards telemedicine [39]. These discrepant findings highlight the important, but often underemphasized, fact that Hispanic individuals in the US comprise a diverse group with substantial differences in country of origin, acculturation, and socioeconomic status. Additionally, as different studies have been done in different periods of the COVID-19 pandemic and focused on different specialties, comparison between studies is challenging. Although our analysis has the benefit of including multiple providers across the country, it is not necessarily a nationally representative sample. It is possible that an analysis of a more nationally representative sample may find different results. However, although exploratory in nature, in our analysis of video telemedicine encounters, Hispanic individuals had a significantly lower rate of video telemedicine encounters than non-Hispanic White individuals. This may reflect the still persistent gap in broadband internet access between Hispanic and non-Hispanic White individuals in the US [26]. The association between primary language and telemedicine utilization was in the same direction as for Hispanic ethnicity for overall utilization, as well as for video visits. Although primary language was treated as a binary variable (English vs. non-English) after multiple imputation to account for missing data, among the approximately 68% of patients who had language data available, only 21.2% of the non-English speakers identified Spanish as their primary language. This suggests that there may be significant diversity in the demographics of the non-English speakers in the study population. Some previous studies have identified non-English primary language as being associated with lower telemedicine utilization [4,24], However, one analysis that examined primarily Spanish speaking patients and other non-English speakers separately noted that primarily Spanish speaking patients had a lower utilization of video telemedicine services [40]. Therefore, as with Hispanic ethnicity, the association between primary language and telemedicine utilization likely varies among different populations. As telemedicine becomes an important part of healthcare delivery in the post-pandemic period, a more granular analysis of both Hispanic ethnicity and language is required to fully understand how different populations utilize telemedicine.

## Limitations

Our analysis has certain limitations. As this is an observational study, the associations noted cannot be assumed to be causal in nature. As the Healthjump database only has data available on completed medical encounters, it cannot be generalized to individuals who had no medical encounters during the pandemic. However, since the rate of telemedicine utilization is similar to those seen in prior studies that included insured patients without any medical encounters, it likely does reflect overall trends in telemedicine utilization in the US during the pandemic. The Healthjump database is not necessarily representative of the overall US patient population. However, it is a large national database with a high proportion of ethnic and racial minority patients, for whom disparities in healthcare access has particularly been an issue. Since patient residence information was limited to 3-digit ZIP code, we were unable to study area-level differences in telemedicine utilization on a more granular level. However, the 3-digit ZIP code level, which is more diverse than some studies that examined state level differences, does provide some insight into sub-state level differences in utilization. Although the main regression model included provider level random intercepts, to account for clustering of patients with the same provider, other important provider level variables such as provider setting and the nature of the telemedicine infrastructure, were not available. Other unmeasured confounders, such as health insurance type and more granular demographic and geographic information, could also play a role in the associations noted. Due to issues of potential misclassification, and lack of data on health insurance type, the primary model did not differentiate between audio only and video telemedicine encounters. However, as a secondary, exploratory analysis, we did examine video telemedicine encounters.

## Conclusions

Approximately one in five patients in a large national database of outpatient encounters had a telemedicine encounter during the COVID-19 pandemic in the US from March through December 2020. However, there were differences in utilization across different sub-groups, with lower odds of receiving telemedicine among older, male, and non-Hispanic Black patients. With ongoing legislative efforts to determine the future of telemedicine after the pandemic, equitable access to telemedicine will require understanding and addressing the particular concerns of different patient populations.

## Supporting information

S1 Appendix Supplementary material. Table 1: Components of the Social Vulnerability Index. Table 2: Current procedural terminology codes to identify outpatient medical encounters. Table 3: Proportion of patients in different race/ethnicity and language sub-groups after multiple imputation. Table 4: Medical comorbidities by telemedicine utilization. Fig 1: Monthly proportion of unique patients with any telemedicine encounters from March to December 2020. Fig 2: Monthly proportion of total outpatient medical encounters that were telemedicine encounters from March to December 2020. Table 5: Mixed effects multivariable logistic regression model coefficients for indicator variables for medical comorbidities.Table 6: Complete case mixed effects multivariable logistic regression model. Table 7: Variance inflation factor. Table 8: Mixed effects multivariable logistic regression model—Outcome: At least one video telemedicine encounter vs. no video telemedicine encounters (among patients with telemedicine encounters).
(DOCX)

## References

[1] Centers for Medicare & Medicaid Services. Medicare Telemedicine Health Care Provider Fact Sheet: Centers for Medicare & Medicaid Services; 2020 [cited 2021 April 8]. Available from: https://www.cms.gov/newsroom/fact-sheets/medicare-telemedicine-health-care-provider-fact-sheet.

[2] Weigel G, Ramaswamy A, L S, Salganicoff A, Cubanski J, Freed M. Opportunities and Barriers for Telemedicine in the U.S. During the COVID-19 Emergency and Beyond. Kaiser Family Foundation [Internet]. 2020 April 8, 2021. Available from: https://www.kff.org/womens-health-policy/issue-brief/opportunities-and-barriers-for-telemedicine-in-the-u-s-during-the-covid-19-emergency-and-beyond/.

[3] Patel SY, Mehrotra A, Huskamp HA, Uscher-Pines L, Ganguli I, Barnett ML. Trends in Outpatient Care Delivery and Telemedicine During the COVID-19 Pandemic in the US. JAMA Intern Med. 2021;181(3):388–91. Epub 2020/11/17. doi: 10.1001/jamainternmed.2020.5928 ; PubMed Central PMCID: PMC7670397. 33196765 PMC7670397

[4] Eberly LA, Kallan MJ, Julien HM, Haynes N, Khatana SAM, Nathan AS, et al. Patient Characteristics Associated With Telemedicine Access for Primary and Specialty Ambulatory Care During the COVID-19 Pandemic. JAMA Netw Open. 2020;3(12):e2031640. Epub 2020/12/30. doi: 10.1001/jamanetworkopen.2020.31640 ; PubMed Central PMCID: PMC7772717. 33372974 PMC7772717

[5] Eberly LA, Khatana SAM, Nathan AS, Snider C, Julien HM, Deleener ME, et al. Telemedicine Outpatient Cardiovascular Care During the COVID-19 Pandemic: Bridging or Opening the Digital Divide? Circulation. 2020;142(5):510–2. Epub 2020/06/09. doi: 10.1161/CIRCULATIONAHA.120.048185 . 32510987 PMC9126131

[6] Healthjump supported EHR integrations. Available from: https://www.healthjump.com/ehr-integrations.

[7] Ziedan E, Simon K, Wing C. Effects of State COVID-19 Closure Policy on Non-COVID-19 Health Care Utilization. National Bureau of Economic Research Working Paper No. 27621. July 2020.

[8] COVID-19 Research Database [cited 2021 April 8]. Available from: https://covid19researchdatabase.org/.

[9] CDC SVI 2018 Documentation. Agency for Toxic Substances and Disease Registry [Internet]. 2020 April 8, 2021. Available from: https://www.atsdr.cdc.gov/placeandhealth/svi/documentation/pdf/SVI2018Documentation-H.pdf.

[10] Bilal U, Tabb LP, Barber S, Diez Roux AV. Spatial Inequities in COVID-19 Testing, Positivity, Confirmed Cases, and Mortality in 3 U.S. Cities: An Ecological Study. Ann Intern Med. 2021. Epub 2021/03/30. doi: 10.7326/M20-3936 . 33780289 PMC8029592

[11] Neelon B, Mutiso F, Mueller NT, Pearce JL, Benjamin-Neelon SE. Spatial and temporal trends in social vulnerability and COVID-19 incidence and death rates in the United States. PLoS One. 2021;16(3):e0248702. Epub 2021/03/25. doi: 10.1371/journal.pone.0248702 ; PubMed Central PMCID: PMC7990180. 33760849 PMC7990180

[12] Carmichael H, Moore A, Steward L, Velopulos CG. Using the Social Vulnerability Index to Examine Local Disparities in Emergent and Elective Cholecystectomy. J Surg Res. 2019;243:160–4. Epub 2019/06/10. doi: 10.1016/j.jss.2019.05.022 . 31177035

[13] 2010 Census Urban and Rural Classification and Urban Area Criteria 2022 [updated October 8, 2021; cited 2022 March 9]. Available from: https://www.census.gov/programs-surveys/geography/guidance/geo-areas/urban-rural/2010-urban-rural.html.

[14] Patel SY, Mehrotra A, Huskamp HA, Uscher-Pines L, Ganguli I, Barnett ML. Variation In Telemedicine Use And Outpatient Care During The COVID-19 Pandemic In The United States. Health Aff (Millwood). 2021;40(2):349–58. Epub 2021/02/02. doi: 10.1377/hlthaff.2020.01786 ; PubMed Central PMCID: PMC7967498. 33523745 PMC7967498

[15] Elixhauser A, Steiner C, Harris DR, Coffey RM. Comorbidity measures for use with administrative data. Med Care. 1998;36(1):8–27. Epub 1998/02/07. doi: 10.1097/00005650-199801000-00004 9431328

[16] Mehrotra A, Jena AB, Busch AB, Souza J, Uscher-Pines L, Landon BE. Utilization of Telemedicine Among Rural Medicare Beneficiaries. JAMA. 2016;315(18):2015–6. Epub 2016/05/11. doi: 10.1001/jama.2016.2186 ; PubMed Central PMCID: PMC4943212. 27163991 PMC4943212

[17] Katz AJ, Haynes K, Du S, Barron J, Kubik R, Chen RC. Evaluation of Telemedicine Use Among US Patients With Newly Diagnosed Cancer by Socioeconomic Status. JAMA Oncol. 2022;8(1):161–3. Epub 2021/11/19. doi: 10.1001/jamaoncol.2021.5784 ; PubMed Central PMCID: PMC8603229. 34792526 PMC8603229

[18] Gareth J, Daniela W, Trevor H, Robert T. An introduction to statistical learning: with applications in R: Spinger; 2013.

[19] Menard S. Collinearity. Applied Logistic Regression Analysis Second Edition (Quantitative Applications in the Social Sciences) Sage Publications, Inc, California2001. p. 75–8.

[20] US Census Bureau. 2019 TIGER/Line ShapefilesTechnical Documentation. 2019. Available from: https://www2.census.gov/geo/pdfs/maps-data/data/tiger/tgrshp2019/TGRSHP2019_TechDoc.pdf.

[21] Medicare telemedicine health care provider fact sheet: Centers for Medicare & Medicaid Services; 2020 [updated March 17, 2021; cited 2021 April 21]. Available from: https://www.cms.gov/newsroom/fact-sheets/medicare-telemedicine-health-care-provider-fact-sheet.

[22] Winter A, Tabor L. Expansion of telehealth in Medicare. September 4 Meeting Presentation. Medicare Payment Advisory Commission. 2020 May 7, 2021 May 7, 2021]. Available from: http://www.medpac.gov/docs/default-source/meeting-materials/medpac_telehealth_presentation_sept_2020_final.pdf?sfvrsn=0.

[23] Campos-Castillo C, Anthony D. Racial and ethnic differences in self-reported telehealth use during the COVID-19 pandemic: a secondary analysis of a US survey of internet users from late March. J Am Med Inform Assoc. 2021;28(1):119–25. Epub 2020/09/08. doi: 10.1093/jamia/ocaa221 ; PubMed Central PMCID: PMC7499625. 32894772 PMC7499625

[24] Chunara R, Zhao Y, Chen J, Lawrence K, Testa PA, Nov O, et al. Telemedicine and healthcare disparities: a cohort study in a large healthcare system in New York City during COVID-19. J Am Med Inform Assoc. 2021;28(1):33–41. Epub 2020/09/01. doi: 10.1093/jamia/ocaa217 ; PubMed Central PMCID: PMC7499631. 32866264 PMC7499631

[25] Walia A, Ravindran S. America’s racial gap and big tech’s closing window. Deutsche Bank Research [Internet]. 2020 April 15, 2021. Available from: https://www.dbresearch.com/PROD/RPS_DE-PROD/PROD0000000000511664/America%27s_Racial_Gap_%26_Big_Tech%27s_Closing_Window.PDF.

[26] Internet/Broadband fact sheet: Pew Reserach Center; 2021 [updated April 7, 2021; cited 2021 April 15]. Available from: https://www.pewresearch.org/internet/fact-sheet/internet-broadband/.

[27] George S, Hamilton A, Baker RS. How do low-income urban African Americans and Latinos feel about telemedicine? A diffusion of innovation analysis. International journal of telemedicine and applications. 2012;2012. doi: 10.1155/2012/715194 22997511 PMC3444862

[28] Banks I. No man’s land: men, illness, and the NHS. BMJ. 2001;323(7320):1058–60. Epub 2001/11/03. doi: 10.1136/bmj.323.7320.1058 ; PubMed Central PMCID: PMC1121551. 11691768 PMC1121551

[29] Himmelstein MS, Sanchez DT. Masculinity impediments: Internalized masculinity contributes to healthcare avoidance in men and women. Journal of health psychology. 2016;21(7):1283–92. doi: 10.1177/1359105314551623 25293967

[30] Pinkhasov RM, Wong J, Kashanian J, Lee M, Samadi DB, Pinkhasov MM, et al. Are men shortchanged on health? Perspective on health care utilization and health risk behavior in men and women in the United States. Int J Clin Pract. 2010;64(4):475–87. Epub 2010/05/12. doi: 10.1111/j.1742-1241.2009.02290.x . 20456194

[31] Thompson AE, Anisimowicz Y, Miedema B, Hogg W, Wodchis WP, Aubrey-Bassler K. The influence of gender and other patient characteristics on health care-seeking behaviour: a QUALICOPC study. BMC Fam Pract. 2016;17:38. Epub 2016/04/03. doi: 10.1186/s12875-016-0440-0 ; PubMed Central PMCID: PMC4815064. 27036116 PMC4815064

[32] Weisman CS, Teitelbaum MA. Women and health care communication. Patient Education and Counseling. 1989;13(2):183–99. doi: 10.1016/0738-3991(89)90060-8 10303324

[33] Misener TR, Fuller SG. Testicular versus breast and colorectal cancer screening: early detection practices of primary care physicians. Cancer Pract. 1995;3(5):310–6. Epub 1995/09/01. 7663550

[34] Mobile fact sheet: Pew Reserach Center; 2021 [updated April 7, 2021; cited 2021 May 7]. Available from: https://www.pewresearch.org/internet/fact-sheet/mobile/.

[35] Call VR, Erickson LD, Dailey NK, Hicken BL, Rupper R, Yorgason JB, et al. Attitudes Toward Telemedicine in Urban, Rural, and Highly Rural Communities. Telemed J E Health. 2015;21(8):644–51. Epub 2015/04/04. doi: 10.1089/tmj.2014.0125 . 25839334

[36] Weber E, Miller SJ, Astha V, Janevic T, Benn E. Characteristics of telehealth users in NYC for COVID-related care during the coronavirus pandemic. J Am Med Inform Assoc. 2020;27(12):1949–54. Epub 2020/09/01. doi: 10.1093/jamia/ocaa216 ; PubMed Central PMCID: PMC7499577. 32866249 PMC7499577

[37] Qian AS, Schiaffino MK, Nalawade V, Aziz L, Pacheco FV, Nguyen B, et al. Disparities in telemedicine during COVID-19. Cancer Med. 2022;11(4):1192–201. Epub 2022/01/07. doi: 10.1002/cam4.4518 ; PubMed Central PMCID: PMC8855911. 34989148 PMC8855911

[38] Drake C, Lian T, Cameron B, Medynskaya K, Bosworth HB, Shah K. Understanding Telemedicine’s "New Normal": Variations in Telemedicine Use by Specialty Line and Patient Demographics. Telemed J E Health. 2022;28(1):51–9. Epub 2021/03/27. doi: 10.1089/tmj.2021.0041 ; PubMed Central PMCID: PMC8785715. 33769092 PMC8785715

[39] Ghaddar S, Vatcheva KP, Alvarado SG, Mykyta L. Understanding the Intention to Use Telehealth Services in Underserved Hispanic Border Communities: Cross-Sectional Study. J Med Internet Res. 2020;22(9):e21012. Epub 2020/09/04. doi: 10.2196/21012 ; PubMed Central PMCID: PMC7499162. 32880579 PMC7499162

[40] Rodriguez JA, Betancourt JR, Sequist TD, Ganguli I. Differences in the use of telephone and video telemedicine visits during the COVID-19 pandemic. American Journal of Managed Care. 2021;27(1). doi: 10.37765/ajmc.2021.88573 33471458 PMC10877492

