# Supplementary material for: Predictors of telemedicine use during the COVID-19 pandemic in the United States–an analysis of a national electronic medical record database
Source: PLoS One. 2022 Jun 29;17(6):e0269535. doi: 10.1371/journal.pone.0269535 (PMC9242497; doi:10.1371/journal.pone.0269535)
Supplement: S1 Appendix — Table 1: Components of the Social Vulnerability Index. Table 2: Current procedural terminology codes to identify outpatient medical encounters. Table 3: Proportion of patients in different race/ethnicity and language sub-groups after multiple imputation. Table 4: Medical comorbidities by telemedicine utilization. Fig 1: Monthly proportion of unique patients with any telemedicine encounters from March to December 2020. Fig 2: Monthly proportion of total outpatient medical encounters that were telemedicine encounters from March to December 2020. Table 5: Mixed effects multivariable logistic regression model coefficients for indicator variables for medical comorbidities.Table 6: Complete case mixed effects multivariable logistic regression model. Table 7: Variance inflation factor. Table 8: Mixed effects multivariable logistic regression model—Outcome: At least one video telemedicine encounter vs. no video telemedicine encounters (among patients with telemedicine encounters). (DOCX) [file pone.0269535.s001.docx]

**S1 Appendix: Supplemental material**

Table 1: Components of the Social Vulnerability Index

Table 2: Current procedural terminology codes to identify outpatient medical encounters

Table 3: Proportion of patients in different race/ethnicity and language sub-groups after multiple imputation

Table 4: Medical comorbidities by telemedicine utilization

Fig 1: Monthly proportion of unique patients with any telemedicine encounters from March to December 2020

Fig 2: Monthly proportion of total outpatient medical encounters that were telemedicine encounters from March to December 2020

Table 5: Mixed effects multivariable logistic regression model coefficients for indicator variables for medical comorbidities

Table 6: Complete case mixed effects multivariable logistic regression model

Table 7: Variance inflation factor

Table 8: Mixed effects multivariable logistic regression model - Outcome: At least one video telemedicine encounter vs. no video telemedicine encounters (among patients with telemedicine encounters)

**Table 1: Components of the Social Vulnerability Index**

| **Socioeconomic Status Index** |
| --- |
| Proportion of residents with income below poverty level |
| Proportion of residents unemployed |
| Median household income |
| Proportion of residents without high school diploma |
| **Household Composition and Disability Index** |
| Proportion of residents who are 65 years of age or older |
| Proportion of residents who are 17 years of age or younger |
| Proportion of civilian residents with a disability |
| Proportion of households that are single-parent households |
| **Minority Status and Language Index** |
| Proportion of residents who are not Non-Hispanic White |
| Proportion of residents who are speaks English “Less than Well” |
| **Housing Type and Transportation Index** |
| Proportion of housing units that are in multi-unit structures |
| Proportion of housing units that are mobile homes |
| Proportion of housing units with more people than rooms |
| Proportion of households with no vehicle |
| Proportion of the population living in group quarters |

All variables derived from the 2019 5-year American Community Survey from the US Census Bureau

**Table 2: Current procedural terminology codes to identify outpatient medical encounters**

| 77427 | 90954 | 92012 | 96127 | 97112 | 97802 | 99473 | G0438 |
| --- | --- | --- | --- | --- | --- | --- | --- |
| 90785 | 90955 | 92013 | 96130 | 97155 | 97803 | 99483 | G0439 |
| 90791 | 90956 | 92014 | 96131 | 97156 | 97804 | 99495 | G0442 |
| 90792 | 90957 | 92507 | 96132 | 97157 | 99201 | 99496 | G0443 |
| 90832 | 90958 | 92508 | 96133 | 97158 | 99202 | 99497 | G0444 |
| 90833 | 90959 | 92521 | 96136 | 97161 | 99203 | 99498 | G0445 |
| 90834 | 90960 | 92522 | 96137 | 97162 | 99204 | 0373T | G0446 |
| 90836 | 90961 | 92523 | 96138 | 97163 | 99205 | S9152 | G0447 |
| 90837 | 90962 | 92524 | 96139 | 97164 | 99211 | 0362T | G0506 |
| 90838 | 90963 | 92601 | 96156 | 97165 | 99212 | G0108 | G0513 |
| 90839 | 90964 | 92602 | 96158 | 97166 | 99213 | G0109 | G0514 |
| 90840 | 90965 | 92603 | 96159 | 97167 | 99214 | G0270 | G2086 |
| 90845 | 90966 | 92604 | 96160 | 97168 | 99215 | G0296 | G2087 |
| 90846 | 90967 | 94005 | 96161 | 97530 | 99354 | G0396 | G2088 |
| 90847 | 90968 | 94664 | 96164 | 97535 | 99355 | G0397 | 97116 |
| 90853 | 90969 | 96110 | 96165 | 97542 | 99406 | G0410 | 97150 |
| 90875 | 90970 | 96112 | 96167 | 97750 | 99407 | G0420 | 97151 |
| 90951 | 92002 | 96113 | 96168 | 97755 | 99441 | G0421 | 97152 |
| 90952 | 92004 | 96116 | 96170 | 97760 | 99442 | G0436 | 97153 |
| 90953 | 97154 | 96121 | 96171 | 97761 | 99443 | G0437 |  |

**Table 3: Proportion of patients in different race/ethnicity and language sub-groups after multiple imputation**

|  | **Any telemedicine encounter** | **No telemedicine encounter** |
| --- | --- | --- |
| Hispanic (any race) | 27.9 (0.08) | 19.2 (0.04) |
| Non-Hispanic Black | 13.2 (0.06) | 13.5 (0.03) |
| Non-Hispanic other race | 4.7 (0.05) | 3.7 (0.02) |
| Non-Hispanic White | 54.1 (0.09) | 63.7 (0.05) |
| English | 77.9 (0.07) | 84.3 (0.04) |
| Non-English | 22.1 (0.07) | 15.7 (0.04) |

Results are presented as proportions (standard errors)

**Table 4: Medical comorbidities by telemedicine utilization**

|  | **Any telemedicine encounter** | **No telemedicine encounter** |
| --- | --- | --- |
| Acquired immune deficiency syndrome | 2,062 (0.5) | 3,894 (0.2) |
| Alcohol abuse | 4,522 (1.0) | 8,561 (0.5) |
| Arthropathies | 11,229 (2.6) | 21,211 (1.4) |
| Blood loss anemia | 1,559 (0.4) | 2,540 (0.2) |
| Cerebrovascular disease | 6,221 (1.4) | 12,316 (0.8) |
| Cerebrovascular disease – sequalae | 1,335 (0.3) | 2,359 (0.2) |
| Chronic pulmonary disease | 41,210 (9.5) | 76,517 (4.9) |
| Coagulopathy | 2,617 (0.6) | 5,172 (0.3) |
| Congestive heart failure | 10,819 (2.5) | 18,856 (1.2) |
| Deficiency anemias | 17,807 (4.1) | 32,083 (2.0) |
| Dementia | 4,881 (1.1) | 8,561 (0.5) |
| Depression | 54,175 (12.5) | 91,006 (5.8) |
| Diabetes with chronic complications | 57,387 (13.3) | 116,321 (7.4) |
| Diabetes without chronic complications | 37,656 (8.7) | 72,186 (4.6) |
| Drug abuse | 7,315 (1.7) | 12,549 (0.8) |
| Hypertension, complicated | 12,194 (2.8) | 24,371 (1.6) |
| Hypertension, uncomplicated | 155,176 (35.9) | 360,039 (23.0) |
| Hypothyroidism | 40,040 (9.3) | 86,023 (5.5) |
| Leukemia | 618 (0.1) | 1,261 (0.1) |
| Liver disease, mild | 8,467 (2.0) | 15,586 (1.0) |
| Liver disease, moderate to severe | 622 (0.1) | 1,101 (0.1) |
| Lymphoma | 956 (0.2) | 1,904 (0.1) |
| Metastatic cancer | 594 (0.1) | 1,246 (0.1) |
| Neurological disorders affecting movement | 5,181 (1.2) | 10,410 (0.7) |
| Obesity | 63,715 (14.7) | 192,416 (12.3) |
| Other neurological disorders | 2,254 (0.5) | 3,768 (0.2) |
| Other thyroid disorders | 7,812 (1.8) | 15,527 (1.0) |
| Paralysis | 2,533 (0.6) | 4,227 (0.3) |
| Peptic ulcer with bleeding | 973 (0.2) | 1,924 (0.1) |
| Peripheral vascular disease | 10,402 (2.4) | 21,909 (1.4) |
| Psychoses | 22,616 (5.2) | 39,604 (2.5) |
| Pulmonary circulation disease | 1,802 (0.4) | 3,105 (0.2) |
| Renal failure, moderate | 16,593 (3.8) | 32,787 (2.1) |
| Renal failure, severe | 4,735 (1.1) | 7,637 (0.5) |
| Seizures and epilepsy | 4,602 (1.1) | 10,097 (0.6) |
| Solid tumor without metastasis, in situ | 450 (0.1) | 868 (0.1) |
| Solid tumor without metastasis, malignant | 8,667 (2.0) | 18,848 (1.2) |
| Valvular disease | 4,653 (1.1) | 10,361 (0.7) |
| Weight loss | 4,323 (1.0) | 8,569 (0.5) |

Results are presented as number of patients and percentage of patients in group (telemedicine or non-telemedicine)

All differences between the two groups are statistically significant at p<0.001

**Fig 1: Monthly proportion of unique patients with any telemedicine encounters from March to December 2020**

**
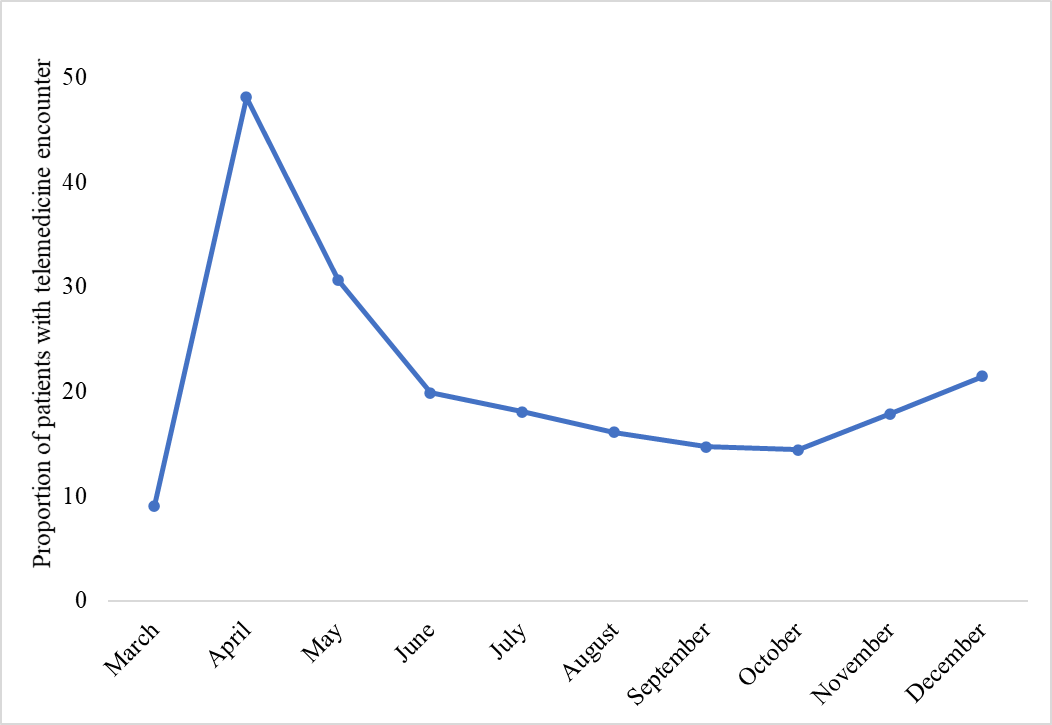
**

**Fig 2: Monthly proportion of total outpatient medical encounters that were telemedicine encounters from March to December 2020**

**
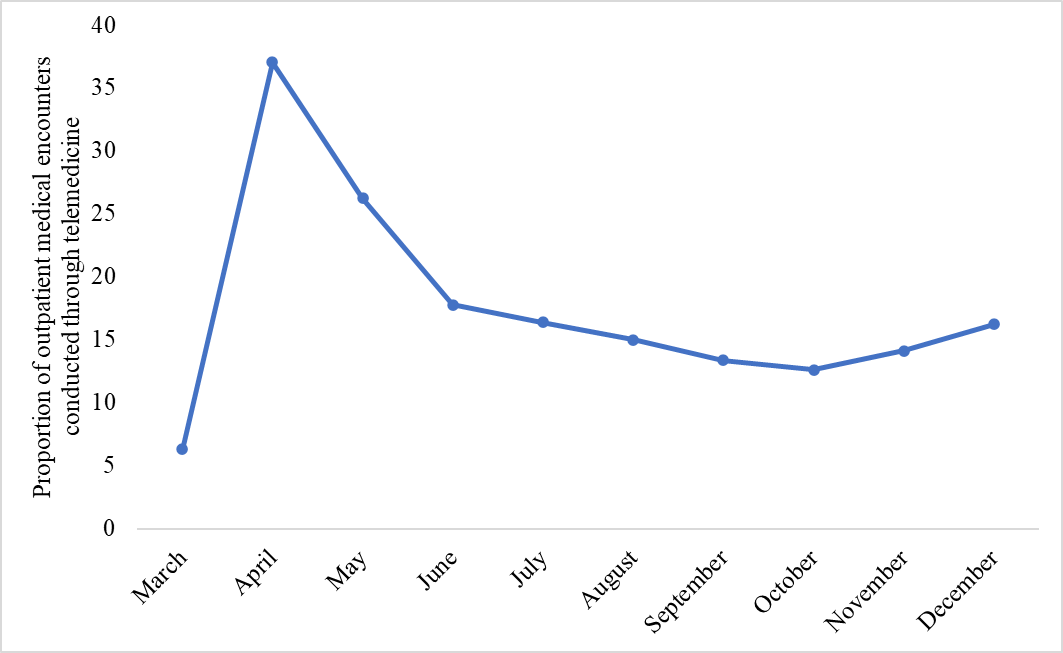
**

**Table 5: Mixed effects multivariable logistic regression model coefficients for indicator variables for medical comorbidities**

| **Comorbidity** | **Odds ratio (95% CI)** | **p-value** |
| --- | --- | --- |
| Acquired immune deficiency syndrome | 1.62 (1.36, 1.92) | <0.001 |
| Alcohol abuse | 1.33 (1.26, 1.41) | <0.001 |
| Arthropathies | 1.68 (1.60, 1.76) | <0.001 |
| Blood loss anemia | 1.49 (1.35, 1.64) | <0.001 |
| Cerebrovascular disease | 1.28 (1.20, 1.36) | <0.001 |
| Cerebrovascular disease – sequalae | 0.95 (0.85, 1.07) | 0.42 |
| Chronic pulmonary disease | 1.73 (1.60, 1.88) | <0.001 |
| Coagulopathy | 1.22 (1.13, 1.32) | <0.001 |
| Congestive heart failure | 1.31 (1.24, 1.38) | <0.001 |
| Deficiency anemias | 1.38 (1.34, 1.43) | <0.001 |
| Dementia | 1.46 (1.35, 1.58) | <0.001 |
| Depression | 2.03 (1.97, 2.09) | <0.001 |
| Diabetes with chronic complications | 1.37 (1.29, 1.46) | <0.001 |
| Diabetes without chronic complications | 1.42 (1.38, 1.47) | <0.001 |
| Drug abuse | 1.64 (1.41, 1.90) | <0.001 |
| Hypertension, complicated | 1.26 (1.14, 1.38) | <0.001 |
| Hypertension, uncomplicated | 1.67 (1.56, 1.79) | <0.001 |
| Hypothyroidism | 1.37 (1.30, 1.45) | <0.001 |
| Leukemia | 1.44 (1.24, 1.68) | <0.001 |
| Liver disease, mild | 1.44 (1.37, 1.51) | <0.001 |
| Liver disease, moderate to severe | 1.11 (0.95, 1.30) | <0.001 |
| Lymphoma | 1.45 (1.28, 1.64) | <0.001 |
| Metastatic cancer | 1.12 (0.93, 1.37) | 0.24 |
| Neurological disorders affecting movement | 1.41 (1.33, 1.49) | <0.001 |
| Obesity | 0.96 (0.93, 0.99) | =0.008 |
| Other neurological disorders | 1.79 (1.62, 1.98) | <0.001 |
| Other thyroid disorders | 1.51 (1.38, 1.66) | <0.001 |
| Paralysis | 1.61 (1.48, 1.75) | <0.001 |
| Peptic ulcer with bleeding | 1.34 (1.20, 1.50) | <0.001 |
| Peripheral vascular disease | 1.14 (1.08, 1.20) | <0.001 |
| Psychoses | 1.79 (1.71, 1.88) | <0.001 |
| Pulmonary circulation disease | 1.22 (1.12, 1.34) | <0.001 |
| Renal failure, moderate | 1.20 (1.12, 1.29) | <0.001 |
| Renal failure, severe | 1.40 (1.19, 1.65) | <0.001 |
| Seizures and epilepsy | 1.32 (1.24, 1.40) | <0.001 |
| Solid tumor without metastasis, in situ | 1.44 (1.22, 1.69) | <0.001 |
| Solid tumor without metastasis, malignant | 1.35 (1.27, 1.43) | <0.001 |
| Valvular disease | 1.25 (1.18, 1.32) | <0.001 |
| Weight loss | 1.37 (1.29, 1.45) | <0.001 |

**Table 6: Complete case mixed effects multivariable logistic regression model**

| **Variable** | **Odds ratio (95% CI)** | **p-value** |
| --- | --- | --- |
| Age | 0.997 (0.99, 1.00) | 0.17 |
| Female | 1.23 (1.12, 1.27) | <0.001 |
| Hispanic (Any race) | 2.15 (1.70, 2.73) | <0.001 |
| Non-Hispanic Black | 1.23 (1.04, 1.45) | 0.01 |
| Non-Hispanic other race | 1.48 (0.88, 2.48) | 0.14 |
| Primarily English speaking | 0.61 (0.54, 0.69) | <0.001 |
| Proportion of residents living in rural areas | 0.99 (0.98, 0.995) | <0.001 |
| Socioeconomic index | 1.01 (1.00, 1.02) | 0.02 |
| Household composition and disability index | 0.995 (0.99, 0.997) | <0.001 |
| Minority status and language index | 0.99 (0.98, 0.993) | <0.001 |
| Housing type and transportation index | 0.999 (0.997, 1.001) | 0.34 |
| Acquired immune deficiency syndrome | 1.72 (1.33, 2.22) | <0.001 |
| Alcohol abuse | 1.33 (1.22, 1.45) | <0.001 |
| Deficiency anemias | 1.34 (1.28, 1.41) | <0.001 |
| Arthropathies | 1.58 (1.48, 1.70) | <0.001 |
| Blood loss anemia | 1.45 (1.27, 1.65) | <0.001 |
| Congestive heart failure | 1.21 (1.13, 1.30) | <0.001 |
| Chronic pulmonary disease | 1.84 (1.69, 2.00) | <0.001 |
| Coagulopathy | 1.20 (1.08, 1.33) | <0.001 |
| Cerebrovascular disease | 1.29 (1.19, 1.40) | <0.001 |
| Cerebrovascular disease – sequalae | 0.92 (0.78, 1.08) | 0.32 |
| Depression | 2.04 (1.95, 2.13) | <0.001 |
| Diabetes with chronic complications | 1.39 (1.31, 1.49) | <0.001 |
| Diabetes without chronic complications | 1.49 (1.42, 1.56) | <0.001 |
| Drug abuse | 1.60 (1.39, 1.86) | <0.001 |
| Hypertension, complicated | 1.29 (1.15, 1.44) | <0.001 |
| Hypertension, uncomplicated | 1.67 (1.56, 1.78) | <0.001 |
| Hypothyroidism | 1.41 (1.33, 1.49) | <0.001 |
| Leukemia | 1.48 (1.20, 1.81) | <0.001 |
| Liver disease, mild | 1.31 (1.23, 1.39) | <0.001 |
| Liver disease, moderate to severe | 1.06 (0.85, 1.33) | 0.59 |
| Lymphoma | 1.60 (1.37, 1.88) | <0.001 |
| Metastatic cancer | 1.12 (0.89, 1.42) | 0.33 |
| Dementia | 1.63 (1.49, 1.79) | <0.001 |
| Neurological disorders affecting movement | 1.45 (1.35, 1.57) | <0.001 |
| Other neurological disorders | 1.64 (1.42, 1.90) | <0.001 |
| Seizures and epilepsy | 1.36 (1.26, 1.48) | <0.001 |
| Obesity | 1.14 (1.09, 1.19) | <0.001 |
| Other thyroid disorders | 1.68 (1.50, 1.89) | <0.001 |
| Paralysis | 1.75 (1.55, 1.98) | <0.001 |
| Peripheral vascular disease | 1.13 (1.06, 1.20) | <0.001 |
| Psychoses | 2.00 (1.86, 2.14) | <0.001 |
| Pulmonary circulation disease | 1.31 (1.15, 1.48) | <0.001 |
| Renal failure, moderate | 1.21 (1.09, 1.34) | <0.001 |
| Renal failure, severe | 1.55 (1.18, 2.05) | 0.002 |
| Solid tumor without metastasis, in situ | 1.41 (1.31, 1.51) | <0.001 |
| Solid tumor without metastasis, malignant | 1.50 (1.21, 1.86) | <0.001 |
| Peptic ulcer with bleeding | 1.46 (1.22, 1.75) | <0.001 |
| Valvular disease | 1.05 (0.96, 1.14) | 0.27 |
| Weight loss | 1.43 (1.32, 1.56) | <0.001 |

Analysis limited to patients with race/ethnicity and language data available

**Table 7: Variance inflation factor**

| **Variable** | **Variance inflation factor** |
| --- | --- |
| Age | 1.23 (0.0002) |
| Female | 1.04 (4.60×10^-5^) |
| Hispanic (Any race) | 1.22 (0.0003) |
| Non-Hispanic Black | 1.18 (0.0002) |
| Non-Hispanic other race | 1.07 (0.001) |
| Primarily English speaking | 1.11 (0.0004) |
| Proportion of residents living in rural areas | 2.69 (0.0004) |
| Socioeconomic index | 4.34 (0.0007) |
| Household composition and disability index | 3.57 (0.0003) |
| Minority status and language index | 2.89 (0.0005) |
| Housing type and transportation index | 1.77 (0.0001) |
| Acquired immune deficiency syndrome | 1.01 (3.78×10^-5^) |
| Alcohol abuse | 1.02 (4.22×10^-6^) |
| Deficiency anemias | 1.04 (0.00004) |
| Arthropathies | 1.01 (1.08×10^-5^) |
| Blood loss anemia | 1.01 (1.08×10^-5^) |
| Congestive heart failure | 1.10 (7.07×10^-6^) |
| Chronic pulmonary disease | 1.04 (1.07×10^-5^) |
| Coagulopathy | 1.02 (7.89×10^-6^) |
| Cerebrovascular disease | 1.04 (9.66×10^-6^) |
| Cerebrovascular disease – sequalae | 1.24 (1.07×10^-5^) |
| Depression | 1.05 (4.6×10^-5^) |
| Diabetes with chronic complications | 1.15 (5.57×10^-5^) |
| Diabetes without chronic complications | 1.07 (6.83×10^-5^) |
| Drug abuse | 1.03 (2.32×10^-5^) |
| Hypertension, complicated | 1.11 (2.84×10^-5^) |
| Hypertension, uncomplicated | 1.31 (0.0001) |
| Hypothyroidism | 1.06 (5.25×10^-5^) |
| Leukemia | 1.00 (2.34×10^-16^) |
| Liver disease, mild | 1.04 (1.70×10^-5^) |
| Liver disease, moderate to severe | 1.03 (5.27×10^-6^) |
| Lymphoma | 1.00 (4.22×10^-6^) |
| Metastatic cancer | 1.03 (4.83×10^-6^) |
| Dementia | 1.02 (1.03×10^-5^) |
| Neurological disorders affecting movement | 1.01 (1.66×10^-5^) |
| Other neurological disorders | 1.00 (6.99×10^-6^) |
| Seizures and epilepsy | 1.01 (0.00003) |
| Obesity | 1.07 (0.0001) |
| Other thyroid disorders | 1.01 (1.15×10^-5^) |
| Paralysis | 1.24 (7.89×10^-6^) |
| Peripheral vascular disease | 1.05 (1.37×10^-5^) |
| Psychoses | 1.03 (2.47×10^-5^) |
| Pulmonary circulation disease | 1.02 (0.00001) |
| Renal failure, moderate | 1.11 (9.72×10^-6^) |
| Renal failure, severe | 1.04 (7.35×10^-5^) |
| Solid tumor without metastasis, in situ | 1.05 (8.76×10^-6^) |
| Solid tumor without metastasis, malignant | 1.00 (3.16×10^-6^) |
| Peptic ulcer with bleeding | 1.00 (8.16×10^-6^) |
| Valvular disease | 1.03 (9.94×10^-6^) |
| Weight loss | 1.01 (1.14×10^-5^) |

Values represent mean and standard deviations across the 10 imputations for each variable.

A Variance inflation factor value >5 was considered evidence for significant multicollinearity.

**Table 8: Mixed effects multivariable logistic regression model - Outcome: At least one video telemedicine encounter vs. no video telemedicine encounters (among patients with telemedicine encounters)**

| **Variable** | **Odds ratio (95% CI)** | **p-value** |
| --- | --- | --- |
| Age | 0.975 (0.972, 0.978) | <0.001 |
| Female | 1.03 (1.001, 1.07) | 0.043 |
| Hispanic (Any race) | 0.56 (0.52, 0.61) | <0.001 |
| Non-Hispanic Black | 0.68 (0.63, 0.74) | <0.001 |
| Non-Hispanic other race | 1.37 (1.16, 1.60) | <0.001 |
| Primarily English speaking | 2.05 (1.83, 2.31) | <0.001 |
| Proportion of residents living in rural areas | 1.01 (1.00, 1.01) | 0.004 |
| Socioeconomic index | 1.00 (0.997, 1.003) | 0.91 |
| Household composition and disability index | 1.01 (1.006, 1.014) | <0.001 |
| Minority status and language index | 1.02 (1.01, 1.02) | <0.001 |
| Housing type and transportation index | 0.994 (0.991, 0.998) | <0.001 |
| Acquired immune deficiency syndrome | 0.402 (0.247, 0.656) | <0.001 |
| Alcohol abuse | 1.141 (0.97, 1.34) | 0.11 |
| Deficiency anemias | 1.01 (0.94, 1.09) | 0.79 |
| Arthropathies | 1.35 (1.23, 1.49) | <0.001 |
| Blood loss anemia | 1.04 (0.83, 1.30) | 0.75 |
| Congestive heart failure | 0.91 (0.84, 0.98) | 0.01 |
| Chronic pulmonary disease | 0.85 (0.81, 0.89) | <0.001 |
| Coagulopathy | 0.95 (0.80, 1.12) | 0.54 |
| Cerebrovascular disease | 0.86 (0.78, 0.95) | 0.003 |
| Cerebrovascular disease – sequalae | 1.18 (0.95, 1.47) | 0.14 |
| Depression | 1.39 (1.31, 1.47) | <0.001 |
| Diabetes with chronic complications | 1.11 (1.04, 1.19) | 0.002 |
| Diabetes without chronic complications | 1.16 (1.10, 1.23) | <0.001 |
| Drug abuse | 1.21 (1.05, 1.40) | 0.01 |
| Hypertension, complicated | 0.64 (0.57, 0.73) | <0.001 |
| Hypertension, uncomplicated | 1.09 (1.03, 1.16) | 0.004 |
| Hypothyroidism | 1.07 (1.02, 1.12) | 0.005 |
| Leukemia | 0.92 (0.66, 1.28) | 0.60 |
| Liver disease, mild | 1.15 (1.02, 1.30) | 0.02 |
| Liver disease, moderate to severe | 0.98 (0.69, 1.38) | 0.90 |
| Lymphoma | 1.16 (0.89, 1.51) | 0.26 |
| Metastatic cancer | 1.01 (0.75, 1.37) | 0.93 |
| Dementia | 0.87 (0.78, 0.98) | 0.02 |
| Neurological disorders affecting movement | 1.00 (0.89, 1.11) | 0.95 |
| Other neurological disorders | 1.71 (1.28, 2.27) | <0.001 |
| Seizures and epilepsy | 0.93 (0.81, 1.06) | 0.25 |
| Obesity | 1.11 (1.05, 1.17) | <0.001 |
| Other thyroid disorders | 1.19 (1.03, 1.38) | 0.02 |
| Paralysis | 0.95 (0.80, 1.13) | 0.55 |
| Peripheral vascular disease | 0.76 (0.70, 0.84) | <0.001 |
| Psychoses | 1.30 (1.19, 1.42) | <0.001 |
| Pulmonary circulation disease | 0.88 (0.74, 1.04) | 0.13 |
| Renal failure, moderate | 0.84 (0.78, 0.89) | <0.001 |
| Renal failure, severe | 1.28 (1.10, 1.49) | 0.002 |
| Solid tumor without metastasis, in situ | 1.01 (0.92, 1.10) | 0.91 |
| Solid tumor without metastasis, malignant | 1.05 (0.69, 1.61) | 0.81 |
| Peptic ulcer with bleeding | 1.11 (0.84, 1.46) | 0.46 |
| Valvular disease | 1.02 (0.90, 1.14) | 0.80 |
| Weight loss | 1.11 (0.97, 1.27) | 0.13 |

Analysis limited to patients with at least one telemedicine encounter during the study period. Telemedicine encounters classified using the following CPT codes: 99441-99443 (audio only), modifier codes GT, GQ, or 95 (audio-video).

Given concerns for misclassification of telemedicine type and lack of health insurance payer information, the results of this model should be considered exploratory.
